# Prevalence and predictors of sexual violence among commercial sex workers in Northern Ethiopia

**DOI:** 10.1186/s12978-015-0036-5

**Published:** 2015-05-23

**Authors:** Mussie Alemayehu, Gebregizabeher Yohannes, Ashenafi Damte, Atsede Fantahun, Kahsu Gebrekirstos, Resom Tsegay, Adina Goldberger, Henock Yebyo

**Affiliations:** Department of Public Health, Mekelle University College of Health Sciences, Mekelle, Ethiopia; Department of Nursing, Abyssinia Medical Sciences, Mekelle, Ethiopia; Department of Nursing, Mekelle University College of Health Sciences, Mekelle, Ethiopia; Department of Nursing, Areaya Kahsu College of Health Sciences, Axum, Ethiopia; Northwestern University, Feinberg School of Medicine, Chicago, USA

**Keywords:** Sexual violence, Commercial sex worker, Mekelle city, Tigray, Ethiopia

## Abstract

**Background:**

Gender-based violence is a natural outgrowth of the stigma and discrimination experienced by commercial sex workers (CSWs) across the globe. In light of this, the current study aimed to describe the prevalence and character of sexual violence, as well as any risk factors for violence, experienced by CSWs in Mekelle City, Northern Ethiopia.

**Methods:**

A cross-sectional study was conducted in Mekelle City in April 2013. 250 CSWs were selected for participation using simple random sampling. Data were collected via a questionnaire instrument. Descriptive statistics and multiple logistic regression analyses were performed using SPSS 20 for Windows.

**Results:**

The overall prevalence of sexual violence among CSWs was 75.6 %. Basic literacy [(AOR = 5.3, 95 % of CI (1.15–25.20)], completion of only elementary school [AOR = 6.9, 95 % of CI (1.55–31.25)], completion of only high school [AOR = 7.9, 95 % of CI (1.65–38.16)], being married [(AOR = 3.8, 95 % CI (1.34–11.09)], engaging in sex work for 1–4 years [(AOR = 5.3, 95 % CI(1.7–16.2)] and drug use [AOR = 5.3, 95 % of CI (1.78–16.21)] were all significant risk factors for sexual violence. CSWs with lower monthly income were also more likely to experience sexual violence; monthly income of 51.2–101.9 USD yielded AOR = 2.4 (95 % CI 1.12–5.37) and monthly income of 102.2–153.1 USD yielded AOR = 7.9 (95 % CI 2.46–25.58), compared to CSWs earning 153.2 USD or more.

**Conclusion:**

The prevalence of sexual violence among CSWs is high. Lower educational attainment, being married, lower monthly income, drug use, and shorter duration of sex work are all risk factors for sexual violence.

## Background

Violence against women has become an increasingly concerning international public health and human rights issue over the past several decades [1]. According to the World Health Organization (WHO), gender-based violence is a global phenomenon that includes physical, psychological and sexual abuse, with the perpetrator often well known to the victim [1, 2]. Sexual violence in specific is tragically pervasive across all cultures, religions, and social classes, and its unfortunate consequences include unintended pregnancy, self-induced abortions, gynecologic problems, sexual dysfunction and sexually transmitted infections (STIs), including HIV. The population of female commercial sex workers (CSWs) in particular is dramatically affected by sexual violence and its many devastating aftermath [2, 3].

Sex workers in many societies are highly stigmatized and subjected to social and political discriminatory treatment [4]. Furthermore, many sex workers consider violence a “normal” part of the job and are not well informed about their rights [4]. As a result, they are often reluctant to report instances of rape and molestation, attempted murder, and beatings or other physical harm [4]. The major contributory factors to the widespread sexual violence against female CSWs are risky sexual behaviors, substance abuse, and patriarchal cultural norms [4]. CSWs are also considered a high-risk group for contracting and spreading HIV and other STIs in the general population [4]. Resulting criminalization of sex work propagates an environment in which violence against sex workers is tolerated, rendering them entirely vulnerable and unable to seek the protection of law enforcement authorities [5].

A 2002 study in Ethiopia demonstrated that 5–20 % of CSWs have been victims of violence [6]. Exclusion of women and girls from the public arena in Ethiopia reinforces gender-based discrimination and renders women socially subordinate to men, setting the stage for this high rate of gender-based violence plaguing the CSW population [7]. In light of this reality, the current study aims to describe the prevalence and predictors of sexual violence experienced by CSWs in Mekelle City, Northern Ethiopia.

## Methods

A community-based cross-sectional study was conducted in Mekelle City in the Tigray region of Northern Ethiopia in April, 2014. According to the recent census conducted by the Central Statistical Agency of Ethiopia (CSA), Mekelle city had a total population of 301,642 in 2011. All CSWs registered in Mekelle City were considered the source population for this study. CSWs who were mentally ill during the data collection period were excluded from the study.

Parameters of 95 % confidence interval (CI), 5 % marginal error, and 20 % estimated frequency of sexual violence (as described in a 2013 study in Adama Town, Ethiopia [3] were used. With an assumption of a 10 % non-response rate and with consideration of the correction formula for finite population, the total sample size computed was 250.

Simple random sampling technique was used to select the study subjects. The city is divided into seven sub-cities. Proportion to size allocation was made for the seven sub-cities based on the number of CSWs found in each sub-city. Based on the sample fraction, women were selected using systematic random sampling. A list of CSWs found in each sub-city was obtained from a CSW outreach program of a save transaction program and was used as a sampling frame. Save transaction program is a program which is owned by save children with its primary role on enhancing the capacity building of CSW including provides loan and screening for STI/HIV/AIDS and provides treatment.

A questionnaire focused on sexual violence as well as CSW demographics and behaviors was distributed to the CSWs included in the study. The questionnaire was prepared in English and translated into the local language (Tigrigna). To confirm consistency of each question, the questionnaire was back-translated into English by a different translator. The questionnaire was adapted from others studies [1, 8, 9] but customized for this context. It queried socio-demographic and economic characteristics of study participants, reproductive history, sexual violence experience, and other behaviors thought to be risk factors for violence. Before the formal onset of the study, the questionnaire was piloted on 5 % of the CSWs in a different area to assess the plausibility of the tool and the estimated length of the interview.

### Data analysis

The data were entered and analyzed using SPSS version 20 for windows (SPSS Inc. version 20, Chicago, Illinois). Descriptive analyses were conducted to estimate the prevalence of sexual violence. The predictors of sexual violence were assessed using multiple logistic regression. The effect size of the predictors was estimated using adjusted Odds Ratio (AOR) for the sample and 95 % CI for ORs for the population effect sizes. A *p*-value of less than 0.05 was considered statistically significant for all tests. Sexual violence among CSWs was defined as experiencing at least one of the following: verbal sexual assault, unwanted genitalia touch, forceful sexual intercourse, request for unusual forms of sexual intercourse, physical harm, and pressure to have sex without a condom.

### Ethics statement

The study protocol was approved by the Institutional Research Review board of Mekelle University College of Health Sciences. Formal letter of permission was obtained from the Mekelle zone health office. Individual verbal consent was obtained from each participant. Participation in the study was voluntary and the participants were free to decline to answer any question. They were informed of the right to withdraw from the study at any time. The participants did not receive any direct benefit or compensation for participating in the study. Participants in this study were exposed only to minimal risk; all collected information was treated as confidential and maintained in a secure location.

## Results

### Socio-demographic characteristics of study population

A total of 250 commercial sex workers participated in the study, indicating a response rate of 100 %. 119 (47.6 %) of the respondents were between the ages of 20 and 24, with a mean age of 24.04 (SD ± 4.3) and an age range of 17 to 47.8 % identified as Ethiopian Orthodox Christians, 79 % identified as ethnically Tigray. 59 (23.6 %) of the respondents were married before they became CSWs. The mean age at marriage was 16.9 (SD ± 2.1), with 18.4 % of the participants reporting marriage before the age of 18. 80 % reported their first episode of sexual intercourse between the ages of 15 and 19, with a mean age of 17 (SD ± 5.7). 10 % of the CSWs reported belonging to agrarian families, while 70.8 % reported being born in an urban area and 96 % reported being raised in an urban area. 10 % of the participants reported an average household monthly income of 51.1–102.2 USD [Table 1].

**Table 1:** Socio-demographic characteristics of respondents in Mekelle city, Northern Ethiopia, 2014

Variables	Frequency	%
Age (years)		
15–19	33	13.20
20–24	119	47.60
25–29	75	30.00
>30	23	9.20
Family occupation		
Farmers	99	39.60
Day laborers	62	24.80
Government employee	42	16.80
Private employee	20	8.20
Self-employed	27	10.80
Ethnicity		
Tigray	175	70.00
Amhara	68	27.20
Other^a^	7	2.80
Religion		
Orthodox Christian	224	89.60
Muslim	17	6.80
Other^b^	9	3.60
Educational status		
Illiterate	26	10.40
Basic literacy	61	24.40
Elementary (1–8)	87	34.80
High school (9–12)	63	25.20
University or college	13	5.20
Dependents		
0	144	57.60
1–3 individuals	78	31.20
4–6 individuals	21	8.40
>6 individuals	7	2.80
Monthly income (USD)		
<51.1	65	26.00
51.2–101.9	109	43.60
102–153.1	65	26.00
≥153.2	11	4.40
Marital status		
Married	59	23.6
Unmarried	191	76.4
Hometown		
Urban	240	96.00
Rural	10	4.00

^a^(Oromo, Afar, Erob) ^b^(Catholic, protestant)

### Reproductive health characteristics of study population

60.4 % of respondents reported nulliparity. 17 % reported history of an STI, with 9.6 % reporting having sought treatment. Gonorrhea was the most common STI reported, accounting for 45.8 % of the total. 27 % of the surveyed CSWs reported a history of at least one elective abortion, with 35.3 % of these women reporting more than one pregnancy termination. 69 % of the respondents acknowledged any type of contraceptive use [Table 2].

**Table 2:** Reproductive health characteristics of respondents in Mekelle city, Northern Ethiopia, 2014

Variables	Frequency	%
Age of marriage [years] (*n* = 59)		
Less than 18	39	66.1
18 and above	20	33.9
Age of first sexual intercourse [years] (*n* = 250)		
Less than 15	25	10
15–19	206	82.40
≥20–24	19	7.6
Parity (*n* = 250)		
Yes	99	39.60
No	151	60.40
History of abortion (*n* = 250)		
Yes	68	27.20
No	182	72.80
History of STI (*n* = 250)		
Yes	43	17.20
No	207	82.80
History of STI treatment (*n* = 43)		
Yes	24	55.8
No	19	44.2
Type of STI (*n* = 250)		
Syphilis	10	41.7
Gonorrhea	11	45.8
Chancroid	03	12.5
History of contraception use (*n* = 250)		
Yes	173	69.2
No	77	30.8

### Social and behavioral characteristics of study population

Cigarette smoking, alcohol consumption, and the chewing of chat were the most common personal habits practiced by this group of CSWs; 114 women (44 %), 170 women (68 %) and 114 women (45.6 %), respectively. 32 % of CSWs reported drug use, and 65.4 % of these women described that the drugs were used for stress relief. 78.8 % of respondents reported working as CSWs for 1–4 years, with lack of money being the primary reason reported for resorting to commercial sex work (43.6 %). 88 % of the women reported occupational condom use, with 70 % reporting that they always had intercourse with a condom. Civil servants were the most common clients reported by the CSWs (45.6 %) [Table 3].

**Table 3:** Behavioral characteristics of respondents in Mekelle city, Northern Ethiopia, 2014

Variable	Frequency	%
Smoking		
Yes	110	44
No	139	56
Alcohol use		
Yes	170	68
No	80	32
Chat chewing		
Yes	114	45.60
No	136	54.40
Drug use		
Yes	81	32.00
No	169	67.60
Reason for drug use		
Stress relief	53	65.4
Peer pressure	13	16
Other^a^	15	18.6
Duration of employment as CSW		
1–4 years	197	78.80
5–9 years	45	18.00
10 or more years	8	3.20
Reason for engaging in sex work		
Shortage of money	109	43.60
Death of family member	20	8.00
Parental divorce	34	13.60
Conflict with family members	18	7.20
Personal divorce	23	9.20
Early marriage	46	18.40
Primary client type		
Merchants	27	10.8
Civil servants	114	45.6
Soldiers	27	10.8
Drivers	30	12.0
Student	52	20.8
Condom use during sexual intercourse		
Yes	220	88.00
No	30	12.00
Consistency of condom use		
Always	158	71.8
Most of the time	62	24.8

^a^Religious reasons, cultural reasons, sexual reasons

### Prevalence of lifetime sexual violence among CSWs

75.6 % of CSWs in this study reported experiencing sexual violence in their lifetime. 55.6 % and 52.4 % of respondents reported being pressured to have sex without a condom and being asked to engage in uncommon modalities of sexual intercourse, respectively. 45.6 % of respondents reported clients inflicting physical harm on them. 60 % of respondents reported experiencing unwanted genitalia touching [Table 4].

**Table 4:** Sexual violence experienced by respondents in Mekelle city, Northern Ethiopia, 2014

Variables	Frequency	%
Any sexual violence		
Yes	189	75.6
No	61	24.4
Pressure to have sex without a condom		
Yes	105	55.6
No	84	44.4
Request for uncommon sexual intercourse		
Yes	99	52.4
No	90	47.6
Physical harm		
Yes	86	45.6
No	103	54.4
Unwanted contact with any sex organ		
Yes	107	56.4
No	82	43.6
Other unwanted touch		
Yes	117	62
No	72	38
Forced sex (rape)		
Yes	82	43.6
No	107	56.4

### Factors associated with sexual violence among CSWs

Multiple logistic regression was utilized to identify significant associations of predictors with sexual violence. Educational status, marital status, drug use, duration of practice in commercial sex work, and monthly income were significant predictors of sexual violence.

Basic literacy and the completion of only elementary or secondary school were significantly associated with sexual violence [(AOR = 5.38, 95 % CI (1.15–25.20), (AOR = 6.96, 95 % CI (1.55–31.25) and (AOR = 7.93, 95 % CI (1.65–38.16)], relative to completion of university-level education, respectively.

With adjustment for other predictors, drug use was significantly associated with sexual violence, with AOR of 5.37 (95 % CI 1.780–16.21). Working for 1–4 years in the commercial sex industry was also significantly associated with sexual violence (AOR = 11. 57, 95 % CI 1.56–85.6), compared to women with longer careers in sex work. Married CSWs were also more likely to experience sexual violence than non-married CSWs (AOR = 3.85, 95 % CI 1.34–11.09). CSWs with a monthly income of 51.2–101.9 USD and with monthly income of 102.2–153.1 USD experienced significantly more sexual violence than CSWs who earned 153.2 USD or more (AOR = 2.44, 95 % CI 1.12–5.37 and AOR = 7.94, 95 % CI 2.46–25.58), respectively [Table 5].

**Table 5:** Factors associated with sexual violence among CSWs in Mekelle city, Northern Ethiopia, 2014

Variables	Violence (*n* = 250)		COR	AOR
	Yes (%)	No (%)		
Educational status				
Illiterate	19(10.10)	7(11.5)	3.17(0.79–12.75	5.56(0.89–34.90)
Basic literacy	43(22.8)	18(29.50)	2.79(0.82–9.45)	5.38(1.15–25.20)*
Elementary school	70(37.00)	17(27.90)	4.8(1.43–16.13)	6.96(1.55–31.25)*
High school	51(27.00)	12(19.90)	4.96(1.41–17.46)	7.93(1.65–38.16)*
College or university	6(3.20)	7(11.50)	1.00	1.00
Marital Status				
Married	52(27.50)	7(11.50)	2.93(1.25–6.85)	3.85(1.34–11.09)*
Unmarried	137(72.50)	54(88.50)	1.00	1.00
Household income (USD)				
<51.1	36(19.00)	29(47.5)	1.00	1.00
51.2–101.9	85(45.00)	24(39.30)	2.85(1.47–5.56)	2.44(1.12–5.37)*
102–153.1	59(31.20)	6(9.80)	7.92(2.99–20.94)	7.94(2.46–25.58)*
≥153.2	9(4.80)	2(3.30)	3.63(0.73–18.12)	3.28(0.54–9.80)
Smoking				
Yes	92(48.70)	19(31.10)	2.09(1.13–3.86)	1.37(0.58–3.25)
No	97(51.30)	42(68.70)	1.00	1.00
Chat chewing				
Yes	94(49.70)	20(32.80)	2.03(1.11–3.72)	1.68(0.26–1.73)
No	95(50.30)	41(67.20)	1.00	1.00
Drug use				
Yes	75(39.70)	6(9.80)	6.03(2.47–14.70)	5.37(1.78–16.21)*
No	114(60.30)	55(90.20)	1.00	1.00
Duration of work as CSW				
1–4 years	159(84.10)	38(62.30)	12.55(2.44–16.64	11.57(1.56–85.6)*
5–9 years	28(14.80)	17(27.90)	4.94(0.89–27.32)	4.57(0.57–39.88)
10 or more years	2(1.10)	6(9.80)	1.00	1.00
Primary client type				
Merchants	21(11.10)	6(9.800	2.19(0.75–6.35)	1.82(0.46–7.24)
Civil servants	93(49.20)	21(34.40)	2.77(1.33–5.76)	2.18(0.89–5.34)
Soldiers	19(10.10)	8(13.10)	1.48(0.55–4.02)	1.21(0.36–4.07)
Drivers	24(12.70)	6(9.80)	2.5(0.87–7.20)	2.68(0.73–9.8)
Students	32(16.90)	20(32.80)	1.00	1.00

*Significantly associated at *p* <0.05

## Discussion

This community-based study has identified the character and prevalence of sexual violence experienced by CSWs in Mekelle city. 75.6 % of the CSWs in this study reported experiencing sexual violence, and education attainment, marital status, drug use, length of time engaged in sex work, and monthly income were significant predictors of sexual violence.

The exclusion of women and girls from the public arena in Ethiopia increases their vulnerability to violence in their communities, reinforces gender-based discrimination, and propagates the social subordination of women [10], and this pattern has been demonstrated in other countries as well [11]. These phenomena manifest especially in the population of CSWs, who are subject to significant additional stigma and discrimination owing to their profession. Previous studies in New York City, India, and Namibia have reported the prevalence of sexual violence in populations of CSWs ranging from 72 to 77 % [11–13]. The finding of this study, a sexual violence prevalence of 75.6 %, is congruent with these. However, a variety of other studies elsewhere in Ethiopia, as well as in Canada and Bahrdar have reported lower rates of sexual violence among CSWs, ranging from 11.4 to 59 %, and higher rates up to 94 % [3, 9, 14, 15]. Variations in the prevalence of violence experienced by CSWs may be related to study methodology or to socio-cultural differences.

Various studies have demonstrated that 46–60 % of CSWs report being raped [16–18], and this study found that 43.6 % of CSWs reported forced sex, which is in line with these previous findings. Rape directly increases sex workers’ risk of infection and gynecologic dysfunction due to vaginal trauma and lacerations, as well as transmission of STIs. It is therefore a particularly brutal form of gender-based violence –both physical and emotionally –with lasting psychological and health consequences for the victim.

CSWs are generally considered a high-risk group for contracting HIV and other STIs and spreading them to the general population [4]. A study in Tanzania demonstrated that 53.7 % of CSWs reported history on an STI, with 89.7 % of these women reporting having unprotected sex with a client while actively infected [19]. This study found an STI rate of 17 % among CSWs, with 90.4 % of these CSWs not seeking treatment from a healthcare facility.

According to the 2011 Ethiopia Demographic and Health Survey, 30 % of married women do not engage in regular paid employment, compared with only 10 % of men [20]. This indicates that men are frequently the sole earner for the family, accentuating their position of social power. Interestingly, married CSWs in this study were more likely to experience sexual violence. This may relate to their increased willingness to submit to subjugation due to their repression at home by their husbands. A 2011 study elsewhere in Ethiopia demonstrated similar findings regarding the prevalence of violence among married CSWs [8].

Lower educational status of CSWs was also a strong predictor of sexual violence. This is likely because more educated CSWs may possess better negotiation, communication and conflict management skills. Furthermore, they may have a better understanding of their rights and legal protections.

Drug use among CSWs may contribute to riskier sexual behavior, and it is therefore not surprising that this study found a significant association between drug use and sexual violence. Furthermore, drug use may render women more vulnerable to violence if they are not in full possession of their faculties while under the influence of drugs, and it may increase their desperation for money in order to fund their habit. This finding is also consistent with previous studies of CSWs in Canada and in Europe [9, 21].

The WHO Sex Work Toolkit: “Community Mobilization” recommends that HIV prevention interventions should employ practical strategies to reduce violence against sex workers [22]. This study recommends several such “practical strategies”, including that policymakers and other leaders establish public awareness activities for the general populace and for CSWs to encourage gender empowerment and discourage gender-based violence. In addition, enhancing sex workers’ access to the protection of law enforcement agents and the legal establishment is another key outlet to reduce sexual violence targeting CSWs, thereby reducing the overall gender-based violence in Northern Ethiopia.

A limitation of this study is that it does not include qualitative data collection. In addition, data related to culturally sensitive issues such as abortion may be invalid, as respondents may not feel comfortable answering these questions in a questionnaire format. Moreover, including the CSW in the registered program may underestimate the result. A future expansion of this research would be to employ qualitative methodology utilizing either individual interviews or focus groups. This may expand constructs in-depth for conceptualizing sexual violence as well as obtain more accurate information on personal matters that women may feel uncomfortable documenting on paper.

## Conclusion

Many CSWs in Mekelle City have been victims of some form of gender-based violence. Lower educational attainment, being married, drug use, shorter duration of employment as a sex worker, and lower monthly income are all risk factors for violence against CSWs.
